# Effects of heat tolerance on the gut microbiota of *Sarcophaga peregrina* (Diptera: Sarcophagidae) and impacts on the life history traits

**DOI:** 10.1186/s13071-023-05973-0

**Published:** 2023-10-17

**Authors:** Lipin Ren, Xiangyan Zhang, Fengqin Yang, Ngando Fernand Jocelin, Yanjie Shang, Qing Wang, Zhuoying Liu, Yadong Guo

**Affiliations:** 1 Department of Forensic Science, School of Basic Medical Sciences, Central South University, Changsha, Hunan China; 2 OE Biotech Co. Ltd, Shanghai, China; 3 Health Law Research Center, School of Law, Central South University, Changsha, Hunan China

**Keywords:** *Sarcophaga peregrina*, Heat tolerance, Microbial community, Developmental cycle

## Abstract

**Background:**

Heat tolerance is a distinct abiotic factor affecting the distribution and abundance of insects. Gut microbiota can contribute to host fitness, thereby increasing resistance to abiotic stress conditions. In this study, *Sarcophaga peregrina* is closely associated with human life in ecological habits and shows remarkable adaptability to daily and seasonal temperature fluctuations. To date, the role of gut microbiota in *S. peregrina* response to heat stress and its influence on the host phenotypic variability remain poorly studied.

**Methods:**

We exposed *S. peregrina* to heat stress at 40 °C for 3 h every day throughout the developmental stages from newly hatched larva to adult, after which gut DNA was extracted from third-instar larvae, early pupal stage, late pupal stage, and newly emerged adults, respectively. Then, 16S rRNA microbial community analyses were performed.

**Results:**

Firstly, we analyzed whether heat stress could have an impact on the life history traits of *S. peregrina* and showed that the growth rate of larvae was higher and the developmental time was significantly shorter after heat stress. We then proposed the role of the gut microbiota in the heat tolerance of *S. peregrina*, which indicated that the bacterial abundance and community structure changed significantly after heat tolerance. In particular, the relative abundance of *Wohlfahrtiimonas* and *Ignatzschineria* was higher in the third-instar larval larvae; the former increased and the latter decreased significantly after heat stress. To further explore the effect of disturbing the microbial community on thermotolerant phenotype, newly hatched larvae were fed with amikacin under heat stress, which indicated that the larval length and the whole developmental cycle was significantly shorter.

**Conclusion:**

This study indicated that *Wohlfahrtiimonas* and *Ignatzschineria* should play an important role in the post-feeding stage under heat stress, but further study is still needed. In general, heat tolerance can affect the gut microbial community structure, which in turn affects the fitness of the host.

**Graphical Abstract:**

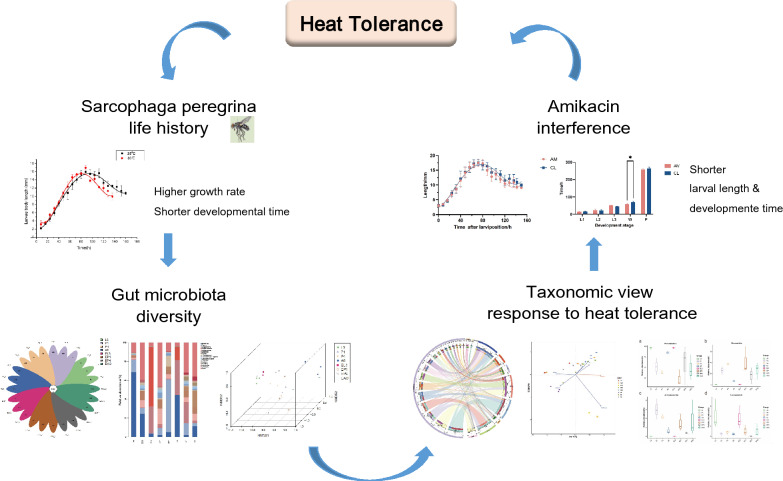

**Supplementary Information:**

The online version contains supplementary material available at 10.1186/s13071-023-05973-0.

## Background

Temperature is the most significant abiotic factor affecting the behavior, life history, physiology, abundance, and distribution of organisms [1]. Insects drive terrestrial ecosystems, and their biology is closely related to ambient temperatures, as they are ectotherms [2]. Temperature fluctuates in the natural environment, and the effects of this variation have been acknowledged in fields as diverse as thermal tolerance physiology, biological control, insect-mediated pollination, disease vector biology, forensic entomology, and simulated climate warming studies [3–5]. In recent years, global warming has led to a substantial increase in the occurrence of extreme heat [6, 7]. In many parts of China, maximum daily temperatures often exceed 40 °C for several hours during the summer [8, 9], posing an extreme threat to the fitness of insects, as this negatively affects insects’ behavior, development, survival, reproduction, and offspring fitness [1, 2]. Insects have an optimal temperature range within which their biological functions are most adaptable, and they may suffer physiological losses that reduce their performance under supra-optimal temperatures. For example, the longer-term effects of heat stress significantly affected the survival, development, and fecundity of the whitefly *Bemisia tabaci* biotype B [10]. It was also reported that he pupae of *Drosophila melanogaster* failed to emerge as adults after heat stress [11], while heat and cold stress induced male sterility in *Drosophila buzzatii* [12]. In transgenic *D. melanogaster*, the proportion of eggs laid by females was significantly reduced after heat stress, suggesting that high temperature has an effect on the insects' offspring [13].

Generally, temperature can affect the composition and diversity of gut microbiota and alter the functional relationship between the host and gut microbiota, which will influence phenotypic variability such as behavioral and life history traits exhibited by the host [14–16]. The ongoing climate change is expected to impose strong selection pressures on the heat tolerance of ectotherms [17], and the gut microbiota can promote thermal tolerance of hosts [18]. Insect midguts provide a favorable environment for microbial colonization, and the midgut bacteria play an important role in the phenotype and fitness of the host [19, 20]. Indeed, recent studies have explored the impact of temperature on the gut microbiota of ectotherms, indicating that the microbiota is sensitive to ambient temperature [21, 22]. For example, *D. melanogaster* reared at low temperature (13 °C) showed the highest abundance of *Wolbachia* in the gut, while that reared at high temperature (31 °C) had the highest abundance of *Acetobacter* (Proteobacteria); in addition, isolating bacterial suspensions from flies reared at low temperatures and then feeding newly fleeced flies resulted in altered heat tolerance in the recipient flies. However, we were not able to link this directly to a change in the host bacterial composition [23]. It was subsequently demonstrated that *D. melanogaster* acclimated in warm conditions showed a higher abundance of *Acetobacter* bacteria and a lower abundance of *Leuconostoc* bacteria (Firmicutes) relative to cold-acclimated flies [24]. Furthermore, recent studies have shown that the diversity and richness of their gut microbiota were decreased when hosts were exposed to higher-temperature conditions [18, 23]. However, the effects of heat stress on the gut microbial community of dipteran flies have been rarely studied.

Therefore, it is worth exploring the relationship between temperature and the gut microbiota in flies to gain a better understanding of how ectotherm species respond to thermal challenges and adapt to new environments. In this study, *Sarcophaga peregrina* was selected (Additional file 1: Fig. S1), which is a member of the Sarcophagidae family (known as flesh fly). This family comprises approximately 3000 described species worldwide, and is closely associated with human life in ecological habits [25]. *Sarcophaga peregrina* is one of the most common flesh flies, with widespread distribution from tropical to subtropical areas of Palearctic, Oriental, and Oceanian regions. The reproductive cycle of *S. peregrina* mostly comprises three definite stages: larva, pupa, and adult. It is well known for adopting the reproductive strategy of ovoviviparity (or ovolarviparity) [26, 27], which can serve as a key indicator in forensic investigations [28], especially in post-mortem interval (PMI) estimation. This method mainly relies on calculating the development time of immature flies that colonize on the decomposed corpses [29], where temperature is the most crucial parameter affecting the development time of flies [26]. So far, most of the developmental time data have been collected at different constant temperatures, but the temperature of the natural environment is never constant [30]. The development time of flies is significantly different under constant temperature versus fluctuating temperature, so neglecting this factor can lead to significant misestimation of the PMI in forensic investigations [31].

As mentioned above, *S. peregrina* shows a prominent capacity for adaptation to daily and seasonal temperature fluctuations and can survive several hours under high temperature. To date, the role of the gut microbiota in *S. peregrina* response to heat stress and its influence on the host phenotypic variability remain poorly studied. In a previous study, we published the annotated chromosome-level genome of *S. peregrina* for the first time, where the availability of the genome (National Center for Biotechnology Information [NCBI] accession no. JABZEU000000000) allowed us to identify potential candidate genes as the reference genome [27]. Here, 40 °C was selected as the optimal temperature of heat stress based on preliminary experiments. We exposed *S. peregrina* to heat stress at 40 °C for 3 h every day throughout the developmental stages from newly hatched larva to adult, and a control group was reared at a constant temperature of 25 °C. The aim of this study was to elucidate the effects of heat stress on the mil community and life history traits of *S. peregrina*.

## Methods

### Cultivation of *S. peregrina* and thermal treatments

Adult specimens of *S. peregrina* were trapped with pork lung bait in Changsha, Hunan Province, China, employing pork lung as a medium for larviposition and larval rearing. In this study, five lines were established and inbred for six generations to reduce genetic variability. In each generation, mating pairs of adults from each line were kept at 25 ± 1 °C and 70 ± 5% relative humidity with a photoperiod regime of 12:12 h light/darkness in an artificial climate chamber (GPL-250A, Tianjin Laboratory Instrument Equipment Co. Ltd., Tianjin, China). Afterwards, the newly hatched larvae were divided into two groups, which were fed at 25 °C and under heat stress at 40 °C for 3 h every day until they emerged as adults, respectively. The sampled larvae were observed to determine the instar based on the number of clefts in the posterior spiracle. The larval stage includes the first, second, and third instar. The third-instar individuals that just jumped into sand were defined as the post-feeding stage. The post-feeding larvae eventually reached metamorphosis, referred to as the pupal period, and finally emerged as adults. Larvae were collected every 8 h until adult emergence (see Additional file 1: Fig. S1). Three replicates were performed.

The duration and intensity of heat stress were based on the duration and intensity of high temperatures in summer, which usually involve a few hours of particularly high temperatures in central China (max temperature 40 °C for approximately 3 h per day for 20 consecutive days) [32]. Thus, the treatments were designed to examine the effects of high temperature (40 °C) on life history parameters using periods of exposure of 3 h per day. Control groups were kept at a constant temperature of 25 °C to allow normal development. To further explore the effects of gut microbiota on the heat tolerance of *S. peregrina*, amikacin was selected as an antibiotic that interferes with the gut bacteria of *S. peregrina*, and was mixed uniformly into the minced meat. Those reared with amikacin-added minced pig lung were recorded as the AM group, and those fed minced meat without amikacin were designated as the CL group (see Additional file 2: Table S1). The larval feeding substrates were placed in the experimental group (heat stress at 40 °C).

### 16S rRNA microbial community analysis

A total of 240 samples were collected in the control group (25 °C) and the experimental group (heat stress at 40 °C), including third-instar larvae in the control group (L3, n=30) and the experimental group (EL3, n=30), as well as early pupal stage in the control group (P1, n=30) and the experimental group (EP1, n=30), late pupal stage in the control group (P4, n=30) and the experimental group (EP4, n=30), newly emerged adults in the control group (A0, n=30) and the experimental group (EA0, n=30). Ten samples were pooled into a biological replicate. Three biological replicates of each group were performed. Total DNA was extracted from pooled guts utilizing the MagPure Soil DNA KF Kit (Magen, Guangdong, China) following the manufacturer's protocol. Polymerase chain reaction (PCR) amplification of the V3–V4 region of the 16S ribosomal RNA (rRNA) gene was selected using the universal bacterial primers (343F: 5′-TACGGRAGGCAGCAG-3′; 798R: 5′-AGGGTATCTAATCCT-3′) [33]. The PCR products were purified using AMPure XP beads (Beckman Coulter, Inc., USA) and quantified with the Qubit dsDNA [double-stranded DNA] Assay Kit (Life Technologies). Sequencing was performed on an Illumina NovaSeq 6000 system (Illumina, San Diego, CA, USA).

Paired-end reads were filtered using DADA2 with the default parameters of QIIME 2 [34, 35]. The representative read of the amplicon sequence variant (ASV) was selected using the QIIME 2 package, and then annotated and blasted against the SILVA database v138 [35, 36]. Alpha diversity and beta diversity were used for estimating the microbial diversity, and were determined using QIIME software. The microbial diversity in samples was estimated using the alpha diversity including the Chao1, Goods_coverage, Shannon, Observed_species, Simpson, and PD_whole_tree indices. The Bray–Curtis distance matrix performed in the R package was used for Bray–Curtis principal coordinates analysis (PCoA) to estimate the beta diversity. Then we further analyzed the significant differences between different groups using analysis of variance (ANOVA) and Kruskal–Wallis statistical tests. The 16S rRNA sequencing and analysis were conducted by OE Biotech Co., Ltd. (Shanghai, China).

## Results

### Effect of heat tolerance on the life history traits

In the control group and the heat tolerance group, there were significant differences in different developmental stages, including larval (first, second, third, and post-feeding stage), pupal stage, emergence time, and total development time, especially in the third instar (see Additional file 2: Table S2). The developmental time for larvae in the heat stress group was about 25 h shorter than that for the control group, while the whole developmental time for the experimental group was about 73 h shorter than that for the control group. Furthermore, the emergence rate decreased significantly after exposure to heat stress, indicating that heat stress had a significant effect on the development of *S. peregrina*.

Based on the statistical analysis of the developmental time mentioned above, this study analyzed the nonlinear regression equation of larval body length and development time (see Additional file 2: Table S3), which showed that the correlation coefficient (*R*^2^) of the fitting equations of the two groups was greater than 0.99, *P* < 0.001. Meanwhile, the relationship between the larval body length and the development time was also determined (see Additional file 1: Fig. S2). The result showed that the body length of the first-, second-, and third-instar larvae increased continuously, and then body length decreased gradually due to the shortening at the post-feeding stage. Compared with the control group, the growth rate of larvae in the heat stress group was higher, and the development time was significantly shorter. The body length of larvae reached the peak earlier, and the maximum value of body length was significantly greater than that in the control group.

### Effect of heat tolerance on the gut bacterial diversity

**Fig. 1:**
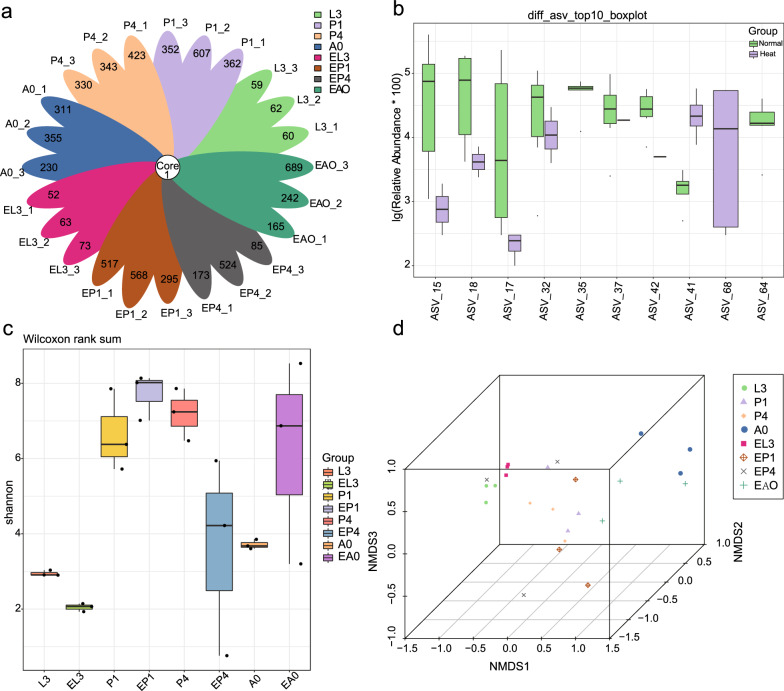
Heat stress induced changes in the microbial communities. **a** The shared and unique number of ASVs in all samples. **b** The relative abundance of ASVs decreased significantly after heat stress (Wilcoxon rank-sum test, *P < 0.05). **c** The alpha diversity was determined by analysis of the Simpson index, with the lower index value in the experimental group indicating that heat stress can reduce the diversity of microbial communities. **d** The analysis of non-metric multidimensional scaling (NMDS) indicated significant differences in microbial communities in different developmental stages of *S. peregrina* after heat stress (*R*^2^ = 0.58153, *P* < 0.001)

Raw reads ranged from 78,174 to 81,591, and clean tags ranged from 39,377 to 70,614 after quality control. Quality filtering and the removal of chimeric sequences yielded 38,232–68,459 valid tags (see Additional file 2: Table S4), indicating that the sequencing data is of high quality. The number of ASVs ranged from 53 to 689 in each sample (Fig. 1a). Heat stress affects the abundance of individual ASVs. A total of 5012 ASVs were identified; 364 ASVs were shared between the two groups, whereas 2294 ASVs were unique to the heat-stressed group and 2354 ASVs were unique to the non-stressed group (see Additional file 1: Fig. S3). The impact of heat stress led to a decrease in abundance of ASVs, among which the relative abundance of 19 ASVs increased significantly after heat stress, in particular two ASVs (ASV_68 and ASV_41) with the highest abundance, whereas 23 ASVs decreased in relative abundance, in particular ASV_15, ASV_18, ASV_17, ASV_35, ASV_32, ASV_37, ASV_42, and ASV_64 (Wilcoxon rank-sum test, *P < 0.05) (Fig. 1b).

The rarefaction curve indicated that the sequencing data were sufficient for all samples, as well as visually showing differences in the richness of species between samples (see Additional file 1: Fig. S5). The species accumulation curve tends to flatten gradually, indicating that the sampling is sufficient, and the samples can reflect the richness of species. The rank abundance curve reflects the abundance and uniformity of species in the sample (see Additional file 1: Fig. S6). The alpha diversity was determined by analysis of the Chao1, Good's coverage, Shannon, observed species, Simpson, and PD [phylogenetic diversity] whole tree indices, where the lower index value in the experimental group indicated that heat stress can reduce the diversity of microbial communities. Furthermore, all index values decreased significantly in the late pupal period throughout the developmental cycle, indicating that changes in gut microbiota after heat stress may have a significant impact on the emergence as adults at the later stage of pupae (Fig. 1c and see Additional file 2: Table S5). This may shed light on why high temperatures lead to lower emergence rates from the perspective of microorganisms. Additionally, beta diversity is the degree of diversity between biological environments, that is, the comparison of differences between samples in different groups. These differences are compared based on the similarity of ASV sequences or the structure of the community. The results showed that there are significant differences in the distribution and structure of gut microbiota. Non-metric multidimensional scaling (NMDS) analysis indicated significant differences in microbial communities between the control group and treatment group, as well as differences at different developmental stages (*R*^2^ = 0.58153, *P* < 0.001) (Fig. 1d).

### Effect of heat tolerance on the distribution of bacterial community

**Fig. 2:**
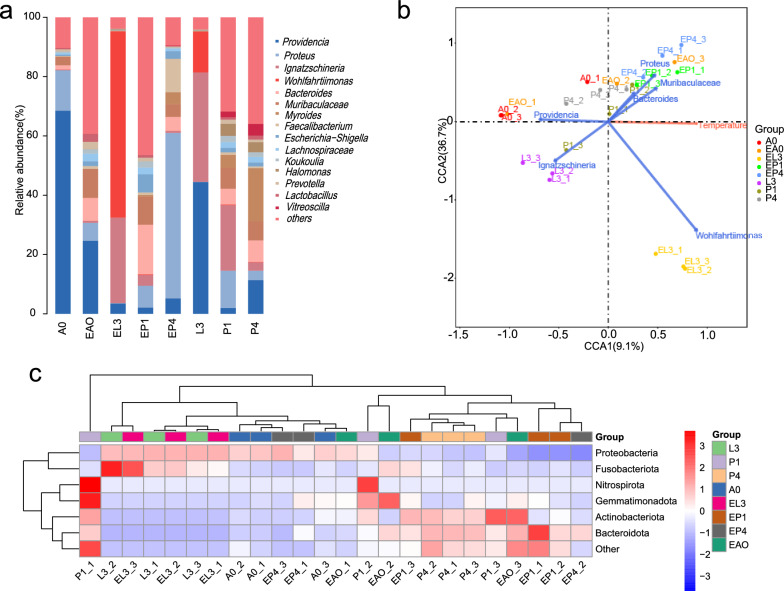
**a** The relative abundance of microbial communities at the genus level. **b** Heatmap showing the relative abundance of top bacterial flora between the heat stress group and the control group analyzed by ANOVA at the phylum level. **c** Canonical correlation analysis (CCA) indicating the relationship between samples, environmental factors (heat stress), and microorganisms, in which the red arrows represent the effect of heat stress and the blue arrows represent different microorganisms. Different colored dots indicated samples from different groups. The angle between microorganisms and environmental factors indicates the positive and negative correlations between species and environmental factors (acute angle: positive correlation; obtuse angle: negative correlation; right angle: no correlation)

We investigated the effect of heat stress on the gut microbiota of *S. peregrina*, revealing that the bacterial composition responded very differently to exposure at 40 °C for 3 h every day, in comparison with the control. At the phylum level, the relative abundance of Firmicutes increased from 10.1 to 18.7% under heat stress, followed by Bacteroidota (from 17.0 to 21.1%). Proteobacteria dominated the bacterial community, with the relative abundance decreasing from 69.1 to 56.6%, followed by Actinobacteriota (from 14.4 to 1.0%). At the genus level, the relative abundance of *Providencia*, which dominated the bacterial community, decreased significantly from 32.8 to 9.1%. The relative abundance of *Ignatzschineria* decreased from 14.7 to 7.7%. The relative abundance of *Proteus* increased from 7.7 to 18.3%, followed by *Wohlfahrtiimonas* (from 3.3 to 14.0%), *Muribaculaceae* (from 3.5 to 7.5%), and *Faecalibacterium* (from 0.4 to 3.5%) (Fig. 2a).

### Taxonomic view across life stages in response to heat tolerance

**Fig. 3:**
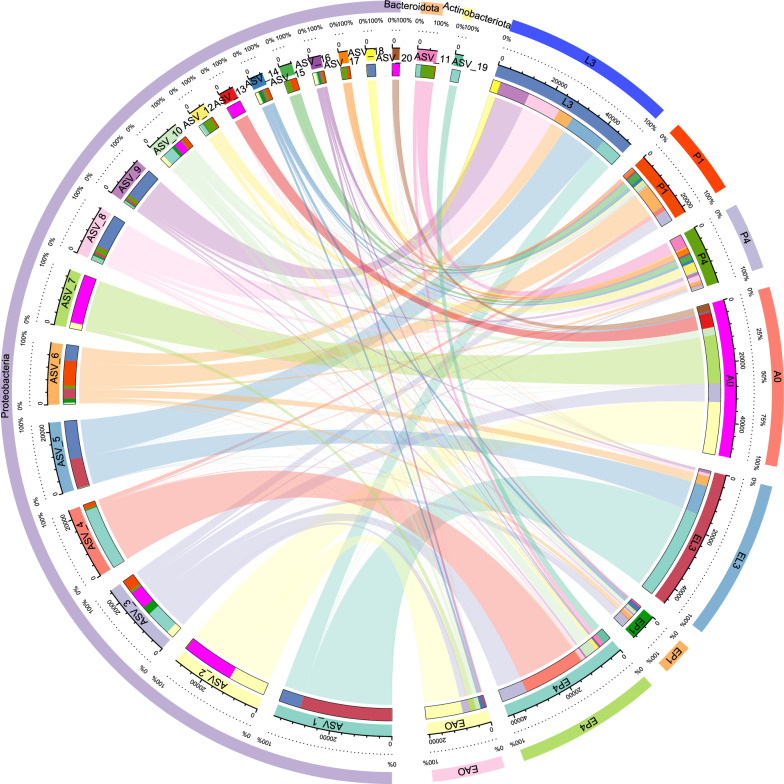
Circos map presenting the corresponding relationship between samples and the microbial community, reflecting the proportion of each dominant microbial community in different groups. The outer and inner circles of the left semicircles represent the relative abundance at the phylum level and the proportion in different groups. The outer and inner circles of the right semicircles represent the different groups and the proportion of species at the phylum level, respectively

In order to further determine whether there were significant differences in different groups, at the phylum level, ANOVA showed that the relative abundance of Proteobacteria, Bacteroidota, and Actinobacteriota at different developmental stages of *S. peregrina* differed significantly under heat stress (Fig. 3 and see Additional file 2: Table S6), while the relative abundance of bacteria at different developmental stages, in particular Proteobacteria, Bacteroidota, and Actinobacteriota, was also demonstrated (Fig. 2b and see Additional file 1: Fig. S6). At the genus level, the relative abundance of *Wohlfahrtiimonas*, *Ignatzschineria*, *Providencia*, *Myroides*, and *Pseudomonas* differed significantly (see Additional file 2: Table S7). Furthermore, these results indicated that at different developmental stages under heat stress, there were significant differences in microbial communities. For example, the relative abundance of *Wohlfahrtiimonas* was higher in the third-instar larvae and increased significantly after heat stress. The relative abundance of *Ignatzschineria* was highest at the third-instar larval stage, decreased significantly after heat stress, and gradually decreased throughout the developmental cycle. Additionally, the relative abundance of *Providencia* was highest in the newly emerged adults but decreased significantly after exposure to heat stress (see Additional file 1: Fig. S7 and Additional file 2: Table S7). Canonical correlation analysis (CCA) further demonstrated the effects of heat stress on microbiota (Fig. 2c).

### Effects of amikacin on the midgut microbes and life history traits

**Fig. 4:**
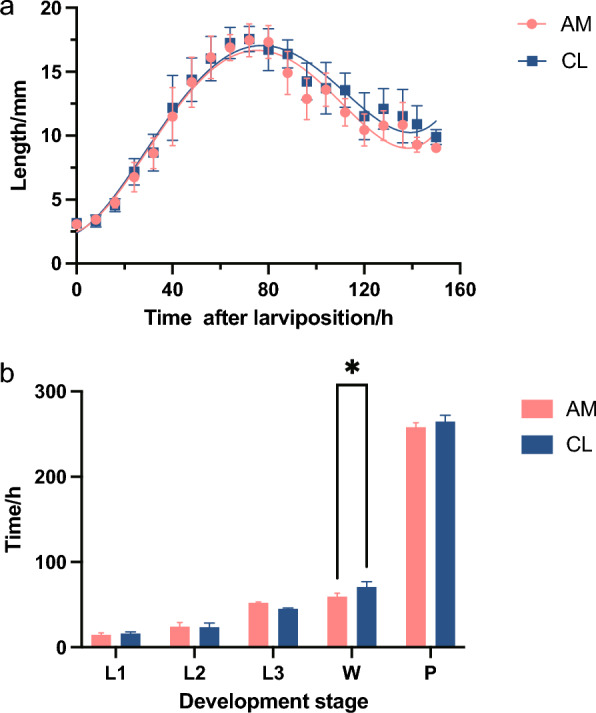
Effects of amikacin on the midgut microbes and life history traits. **a** The effects of amikacin on the length of larvae. The larval length in the AM group was significantly shorter than that in the CL group, after entering the third-instar larval stage. **b** The effect of amikacin on the whole developmental cycle, demonstrating that the developmental time in the AM group was shorter than that in the CL group

Amikacin is an aminoglycoside with strong action against aerobic Gram-negative bacteria. To further explore the effect of microbial community changes on life history traits, newly hatched larvae were fed with amikacin to disturb the gut microbiota, and the larval length in the AM group was significantly shorter than that in the CL group after entering the third-instar larval stage (Fig. 4a). Moreover, we investigated the effect of amikacin on the whole developmental cycle, demonstrating that the developmental time in the AM group was shorter than that in the CL group; in particular, the difference was more significant during the post-feeding stage (*P* < 0.01) (Fig. 4b and see Additional file 2: Table S8). The results confirmed that amikacin can be used to explore the effect of heat stress on larval gut bacteria, but more appropriate concentrations should be further investigated. Furthermore, it can be tentatively inferred that Gram-negative bacteria, which are the main targets of amikacin, play an important role in the resistance of larvae to the adverse effects caused by heat stress.

## Discussion

With the intensification of global warming, the fitness of insects has been seriously affected [24, 37]. As a holometabolous insect, *S. peregrina* undergoes larval–pupal metamorphosis to emerge into adults during the life cycle, and temperature is a key abiotic factor affecting metamorphosis [38, 39]. This study showed that heat stress had a significant effect on the life history traits of *S. peregrina*, in particular with respect to the developmental time and larval body length. Gut microbiota can promote the adaptation of host physiology, resulting in increased resistance under abiotic stressful conditions [14, 19, 21]. Insect midgut microbial symbionts have been considered as an integral component in thermal adaptation due to their differential thermal sensitivity. Altered midgut microbial communities can influence both insect physiology and competence for important vector-borne pathogens [19, 40]. Therefore, we hypothesized that significant changes in the midgut microbiota in *S. peregrina* are associated with heat stress. In this study, we investigated the physiological changes coupled with thermal resistance and life history traits of *S. peregrina* reared at high temperatures. The larva-to-adult developmental time was significantly affected by temperature exposure. The rate of development was relatively fast under heat stress at 40 °C compared with constant temperature at 25 °C. Moreover, we explored the relationship between the gut microbiota and the thermal physiology of *S. peregrina*. These results suggest that heat stress alters the composition and diversity of the gut microbiota. In general, temperature-induced changes in the gut microbiome may impact host fitness, including effects on colonization resistance in the gut, host energy and nutrient assimilation, and host life history traits [22]. Warm conditions lead to a decrease in ASV number (richness) and diversity of the gut microbiota in ectotherms [41, 42]. In this study, we found that the relative abundance of ASVs increased significantly after heat stress. Alpha diversity, determined by analysis of Chao1 and Shannon indices, indicated that microbial community abundance changed significantly after heat stress. PCoA indicated significant differences in microbial communities under heat stress as well. To further identify the specific bacterial communities of *S. peregrina*, Wilcoxon analysis showed that almost all of the top 10 bacterial genera in relative abundance decreased significantly after heat stress, indicating that such stress can negatively affect host fitness.

The composition of the gut microbiota varies substantially across invertebrate animal species but tends to display higher relative abundance of Proteobacteria than gut-microbiota composition in vertebrates. Variation in ambient temperature has been associated with changes in the composition of the gut microbiota in diverse invertebrate lineages [22]. In insects, increases in temperature have been associated with increased relative abundance of Proteobacteria. Developmental temperature has been shown to impact the composition of the gut microbiota of fruit flies, with higher temperatures leading to increased abundance of *Acetobacter* and Proteobacteria [23]. A previous study found that conventional flies are more tolerant to high temperatures than germ-free flies, suggesting that the gut microbiota has a positive influence on the heat tolerance of *Drosophila subobscura* [24]. This may be due to the effect of the gut microbiota on the nutritional status of the host, which in turn determines the growth and development of ectotherms [23, 43, 44]. In general, these studies have used thermal acclimation to evaluate changes in the gut microbiota composition, and it was found that at warm temperatures, bacteria of the phylum Firmicutes gradually decreased in ectotherms of vertebrates [41], whereas warm acclimatization led to an increase in the relative abundance of Proteobacteria in invertebrate ectotherms [23]. We found that in the dominant microbial community, the relative abundance of Firmicutes significantly increased after heat stress, followed by Bacteroidota dominating the bacterial community, and the relative abundance of Proteobacteria and Actinobacteriota decreased dramatically. The relative abundance of *Wohlfahrtiimonas* was higher in the third-instar larvae and increased significantly after heat stress. The relative abundance of *Ignatzschineria* was highest at the third-instar larval stage, decreased significantly after heat stress, and gradually decreased throughout the developmental cycle. Meanwhile, newly hatched larvae that were fed with amikacin to disturb the gut microbiota entered the post-feeding stage prematurely. The results indicated that *Wohlfahrtiimonas* and *Ignatzschineria* play an important role in the larva entering the feeding stage under heat stress [45], with the former positively correlated with heat stress and the latter negatively correlated. Additionally, the relative abundance of *Providencia* was highest in the newly emerged adults but decreased significantly after exposure to heat stress. These results are generally consistent with previous studies; for example, increased temperature led to the enrichment of specific taxa in *Aedes aegypti*, and the top five core phyla in relative abundance were *Actinobacteria*, followed by *Bacteroidetes*, *Cyanobacteria*, *Firmicutes*, and *Proteobacteria* [46].

Previous studies have demonstrated that bacterial diversity and abundance decline when hosts are exposed to a thermal environment, as temperature induces changes in the composition and diversity of the gut microbiota, which may have a dramatic effect on the phenotype and fitness of the host [47], suggesting that the gut microbiota is sensitive to ambient temperature. Indeed, thermal acclimation can affect the taxonomical abundance, diversity, and community structure of the gut microbiota of *D. melanogaster*, with higher temperatures leading to decreased abundance of *Leuconostoc* (Firmicutes), and increased abundance of *Acetobacter* bacteria (*Proteobacteria*) may be caused by providing more optimal growth conditions for acetic acid bacteria [23, 41]. Effects of temperature on the relative abundance of Proteobacteria lineages have also been observed in worms (*Caenorhabditis elegans*), where worms reared at higher temperatures displayed increased relative abundance of *Agrobacterium* and Proteobacteria, and a corresponding decrease in the relative abundance of *Sphingobacterium*, a genus of *Bacteroidetes* [48]. Consistent with previous studies on *D. melanogaster*, the composition and diversity of microbiota of flies developed at different temperatures were simple and consisted mainly of three genera: *Acetobacter*, *Wolbachia*, and *Leuconostoc* [49, 50]. In addition, *D. melanogaster* acclimated in warm conditions showed a higher abundance of *Acetobacter* and a lower abundance of *Leuconostoc* [23]. Here, we found that thermal acclimation led to an increase in the relative abundance of *Proteus*, which dominated the bacterial community, whereas the relative abundance of *Providencia* decreased significantly. Moreover, the higher abundance of *Wolbachia* in flies developed at higher temperatures supports a key involvement of temperature in regulating the presence of this genus in flies and suggests that *Wolbachia* have an impact on host temperature tolerance [23]. The influence of *Wolbachia* on the level and biosynthesis of octopamine depends on the endosymbiont genotype [51]. Moreover, the influence of the *Wolbachia* genotype on *Drosophila* survivability under heat stress correlates with changes in catecholamine (dopamine and octopamine) metabolism in the latter [52]. It is known that catecholamines are stress hormones in insects, which allows us to suppose that the influence of *Wolbachia* on host adaptability could be mediated through changes in the latter's catecholamine metabolism [52, 53].

## Conclusions

In general, heat stress can have an impact on the life history traits of *S. peregrina*. We further found that heat tolerance can affect the gut microbial community structure, which in turn affects the fitness of *S. peregrina*. *Wohlfahrtiimonas* and *Ignatzschineria* should play an important role in the process of metamorphosis after heat stress. To further explore the effect of microbial community disturbance on thermotolerance phenotype, amikacin was fed to newly hatched larvae under heat stress, which indicated that the larval length and the whole developmental cycle were significantly shortened. However, 16S sequencing and interference verification were not performed for the intestinal flora of samples in the antibiotic feeding group under heat stress, and thus further study is still needed.

### Supplementary Information

**Additional file 1. Fig. S1**: A female adult specimen of *S. peregrina*. **Fig. S2**: The relationship between the larval body length and the developmental time is shown. **Fig. S3**: This study showed the shared and unique number of ASVs in the control group and heat stress group, indicating that heat stress induced changes in the abundance of individual ASVs. **Fig. S4**: Rarefaction curves were used to calculate indices based on Good's coverage. **Fig. S5**: The rank abundance curve reflects the abundance and uniformity of species in the sample. **Fig. S6**: The relative abundance of top bacterial flora between the heat stress group and the control group analyzed by ANOVA at the phylum level. **Fig. S7**: The relative abundance of top bacterial flora between the heat stress group and the control group analyzed by ANOVA at the genus level.

**Additional file 2. Table S1**: Feeding substrate preparation. **Table S2**: Development time of *S. peregrina* under heat stress (h) Mean (± SD). **Table S3**: The nonlinear regression equation of larval body length and development time. **Table S4**: Summary statistics of tags during developmental stages in response to heat stress. **Table S5**: The effects of heat stress on bacterial alpha diversity of *S. peregrina* across life stages. **Table S6**: The relative abundance of top bacterial flora between the heat stress group and the control group analyzed by ANOVA at the phylum level. **Table S7**: The relative abundance of top bacterial flora between the heat stress group and the control group analyzed by ANOVA at the genus level. **Table S8**: Mean (± SD) development duration (h) with different treatment methods.

## Data Availability

Raw sequencing data of 16S rRNA have been deposited in the Sequence Read Archive (SRA) database of the National Center for Biotechnology Information (NCBI) via accession numbers SRR17717693–SRR17717722 with the Bioproject ID PRJNA799843.
