# Supplementary material for: Effects of heat tolerance on the gut microbiota of Sarcophaga peregrina (Diptera: Sarcophagidae) and impacts on the life history traits
Source: Parasit Vectors. 2023 Oct 17;16:364. doi: 10.1186/s13071-023-05973-0 (PMC10580603; doi:10.1186/s13071-023-05973-0)
Supplement: Supplementary file 1 — Additional file 1. Fig. S1: A female adult specimen of S. peregrina. Fig. S2: The relationship between the larval body length and the developmental time is shown. Fig. S3: This study showed the shared and unique number of ASVs in the control group and heat stress group, indicating that heat stress induced changes in the abundance of individual ASVs. Fig. S4: Rarefaction curves were used to calculate indices based on Good's coverage. Fig. S5: The rank abundance curve reflects the abundance and uniformity of species in the sample. Fig. S6: The relative abundance of top bacterial flora between the heat stress group and the control group analyzed by ANOVA at the phylum level. Fig. S7: The relative abundance of top bacterial flora between the heat stress group and the control group analyzed by ANOVA at the genus level. [file 13071_2023_5973_MOESM1_ESM.doc]

**Supplementary Data File 1**

**Fig. S1** A female adult specimen of *S. peregrina*.

**Fig. S2** The relationship between the larval body length and the developmental time was shown.

**Fig. S3** This study showed the shared and unique number of ASVs in the control group and heat stress group, indicating that heat stress induced changes in the abundance of individual ASVs.

**Fig. S4** Rarefaction curve were used to calculate indices based on goods coverage.

**Fig. S5** The rank abundance curve reflects the abundance and uniformity of species in the sample.

**Fig. S6** The relative abundances of top bacterial flora between heat stress group and the control group analyzed by ANOVA at phylum level.

**Fig. S7** The relative abundances of top bacterial flora between heat stress group and the control group analyzed by ANOVA at genus level.


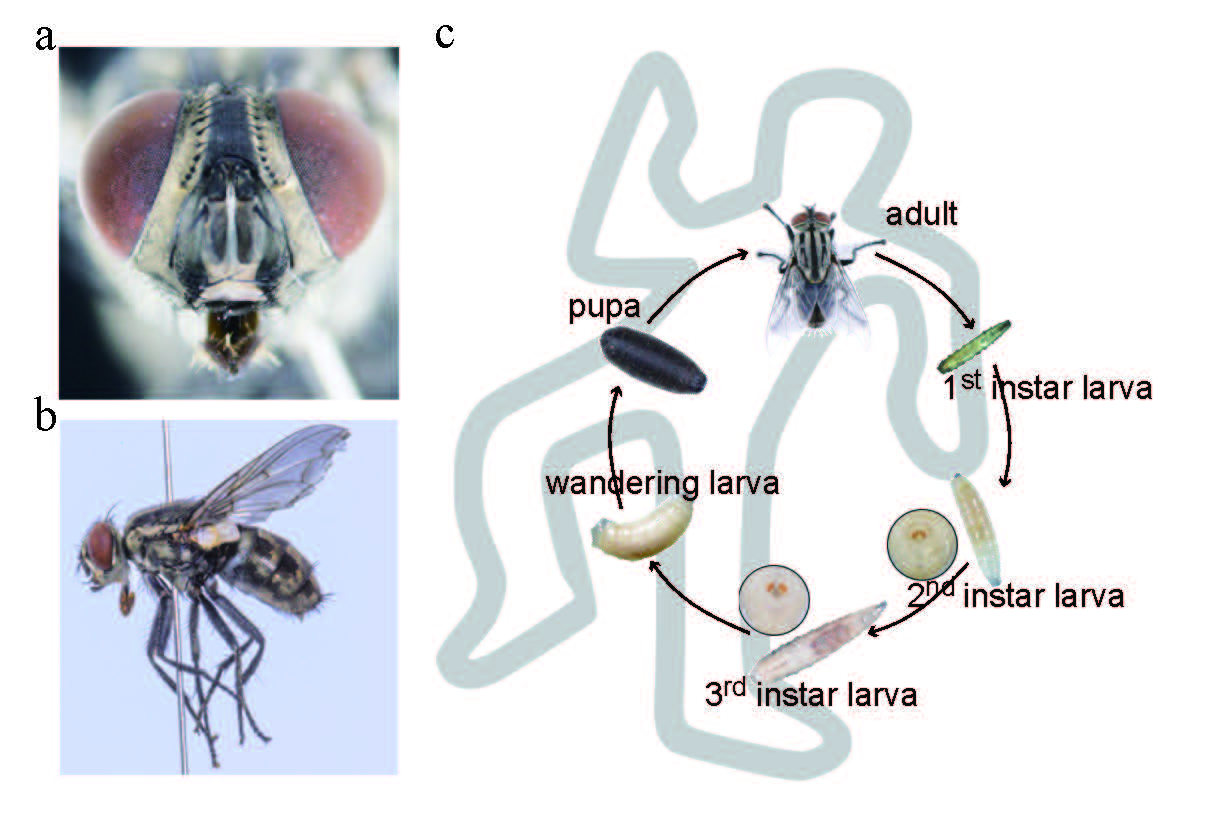


**Fig. S1** A female adult specimen of *S. peregrina*. (a) frontal image; (b) profile image; (c) The life cycle.


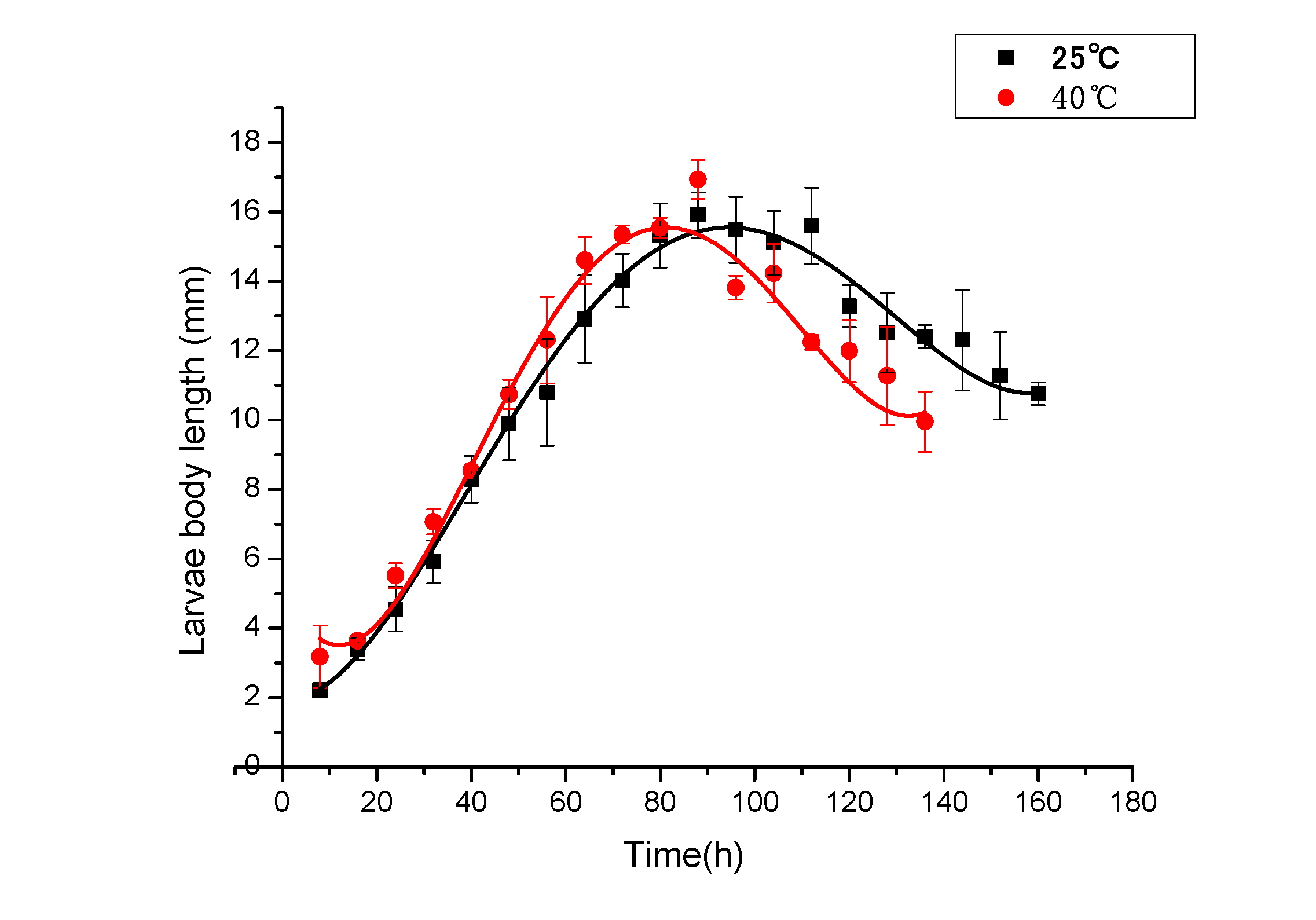


**Fig. S2** The relationship between the larval body length and the developmental time was shown.


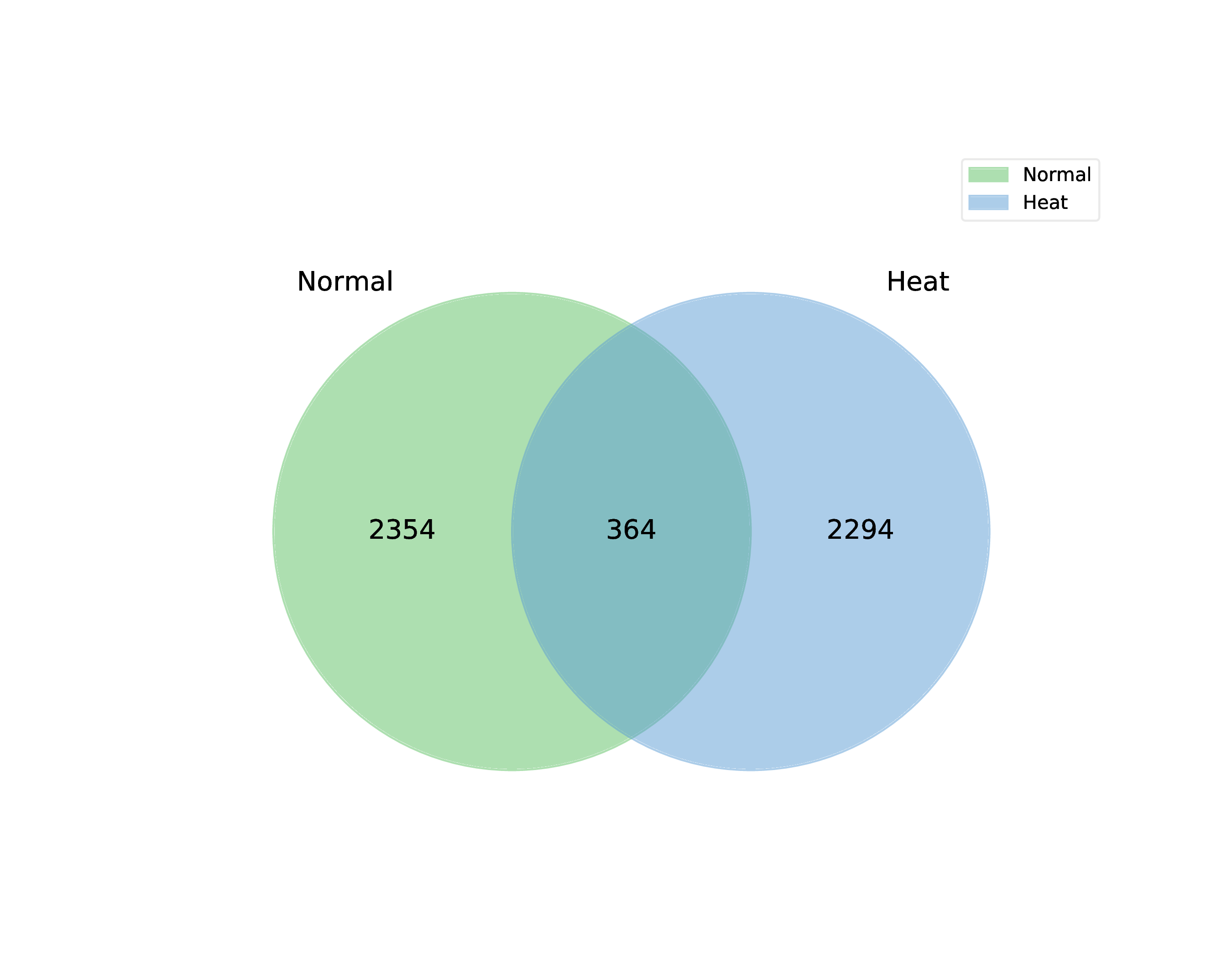


**Fig. S3** This study showed the shared and unique number of ASVs in the control group and heat stress group, indicating that heat stress induced changes in the abundance of individual ASVs.


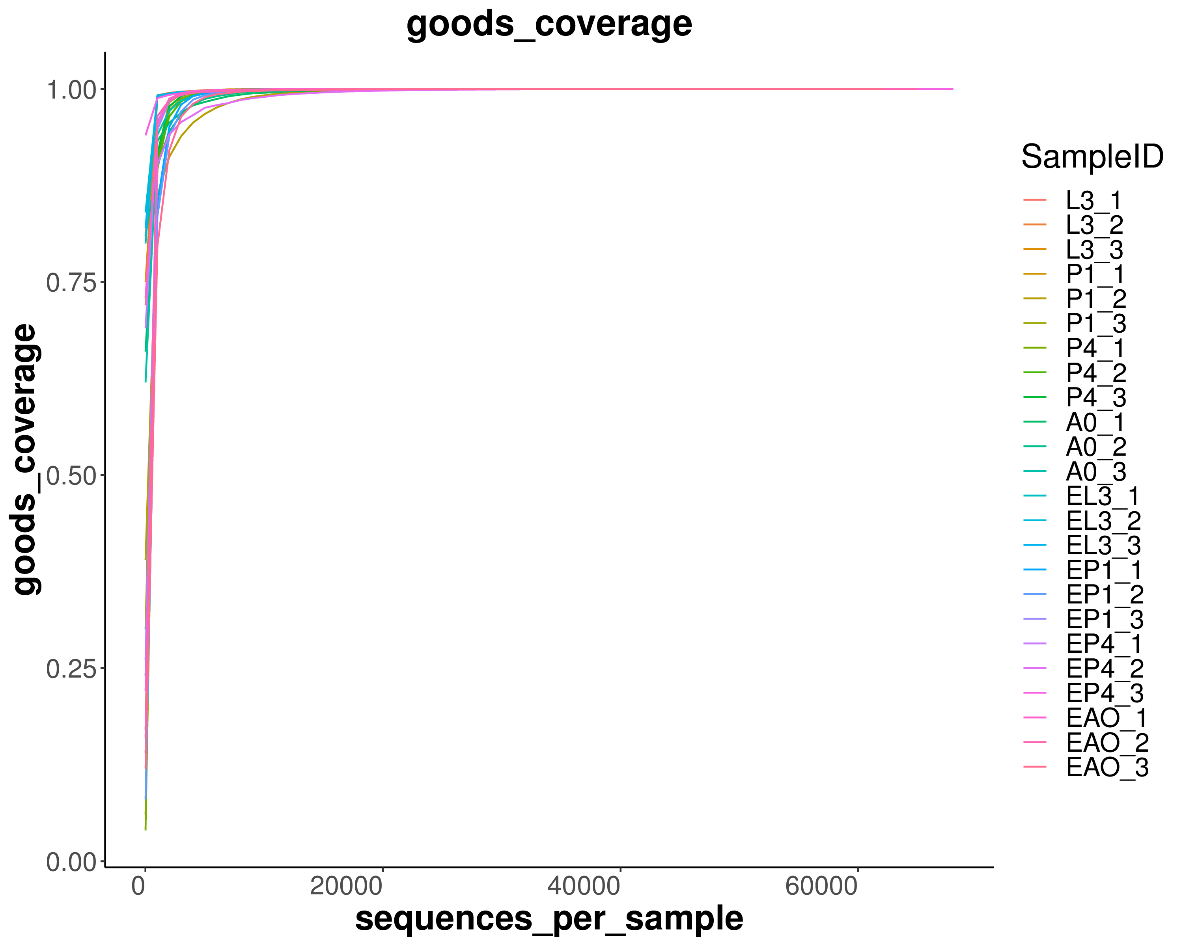


**Fig. S4** Rarefaction curve were used to calculate indices based on goods coverage.


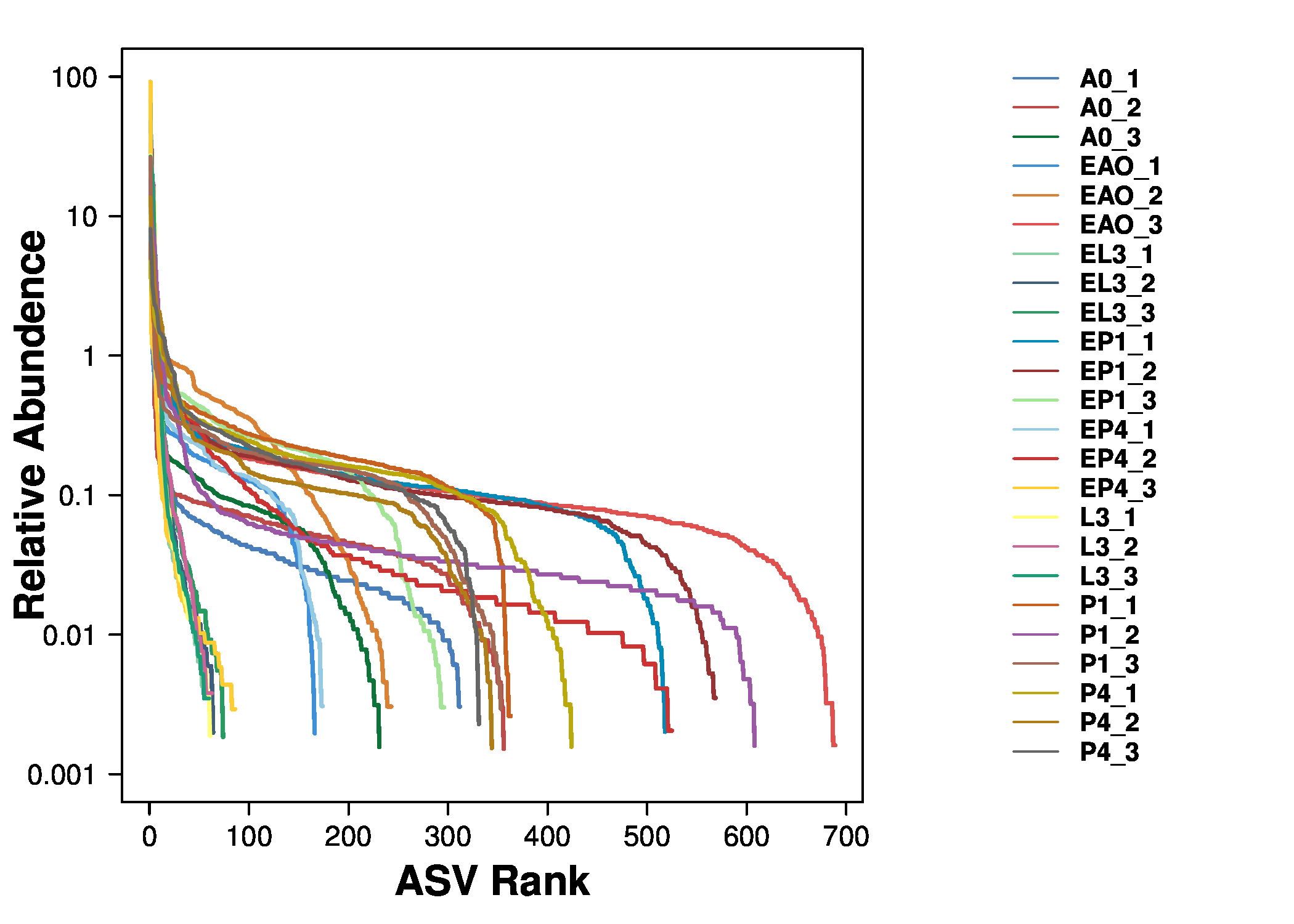


**Fig. S5** The rank abundance curve reflects the abundance and uniformity of species in the samples.


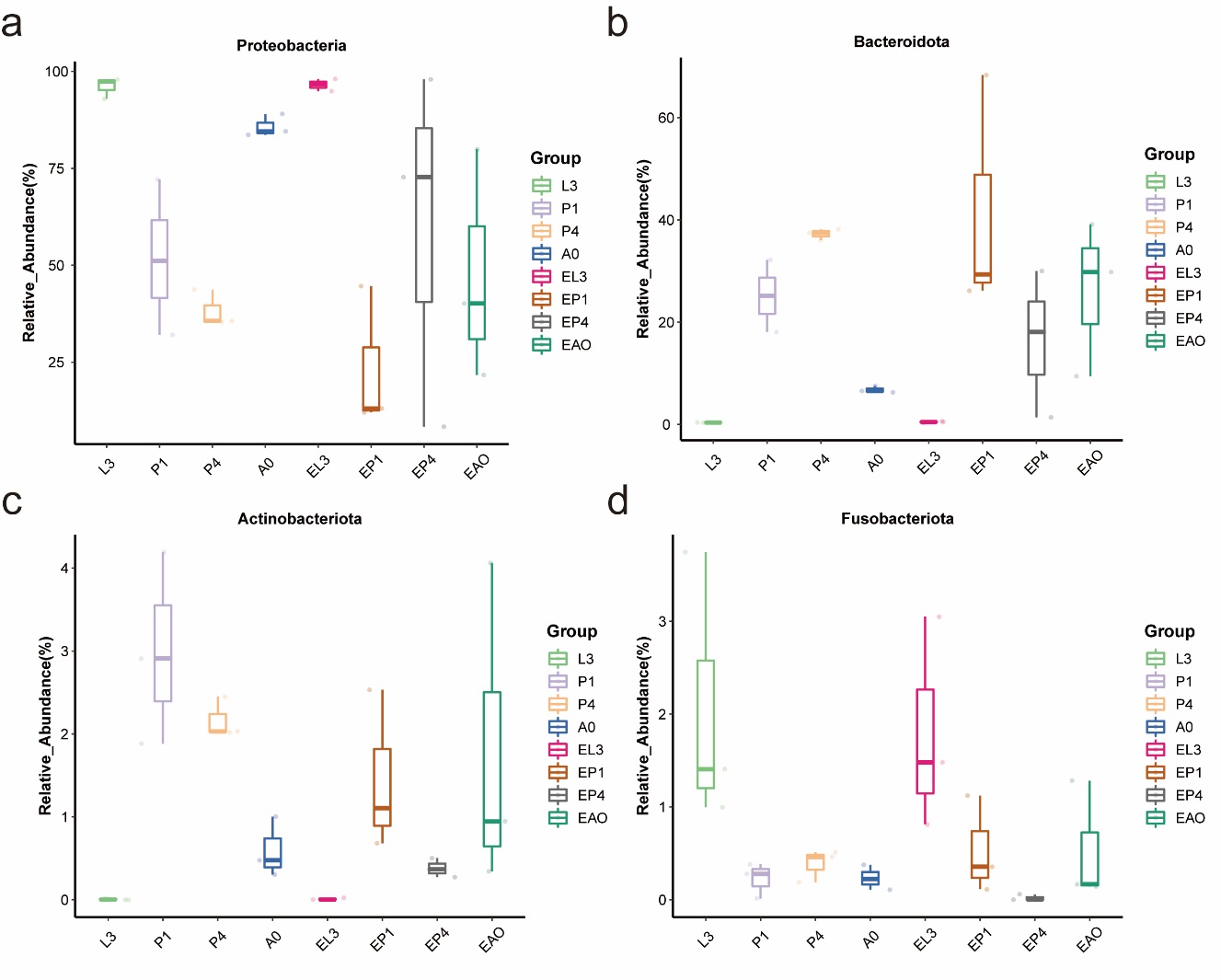


**Fig. S6** The relative abundances of top bacterial flora between heat stress group and the control group analyzed by ANOVA at phylum level.


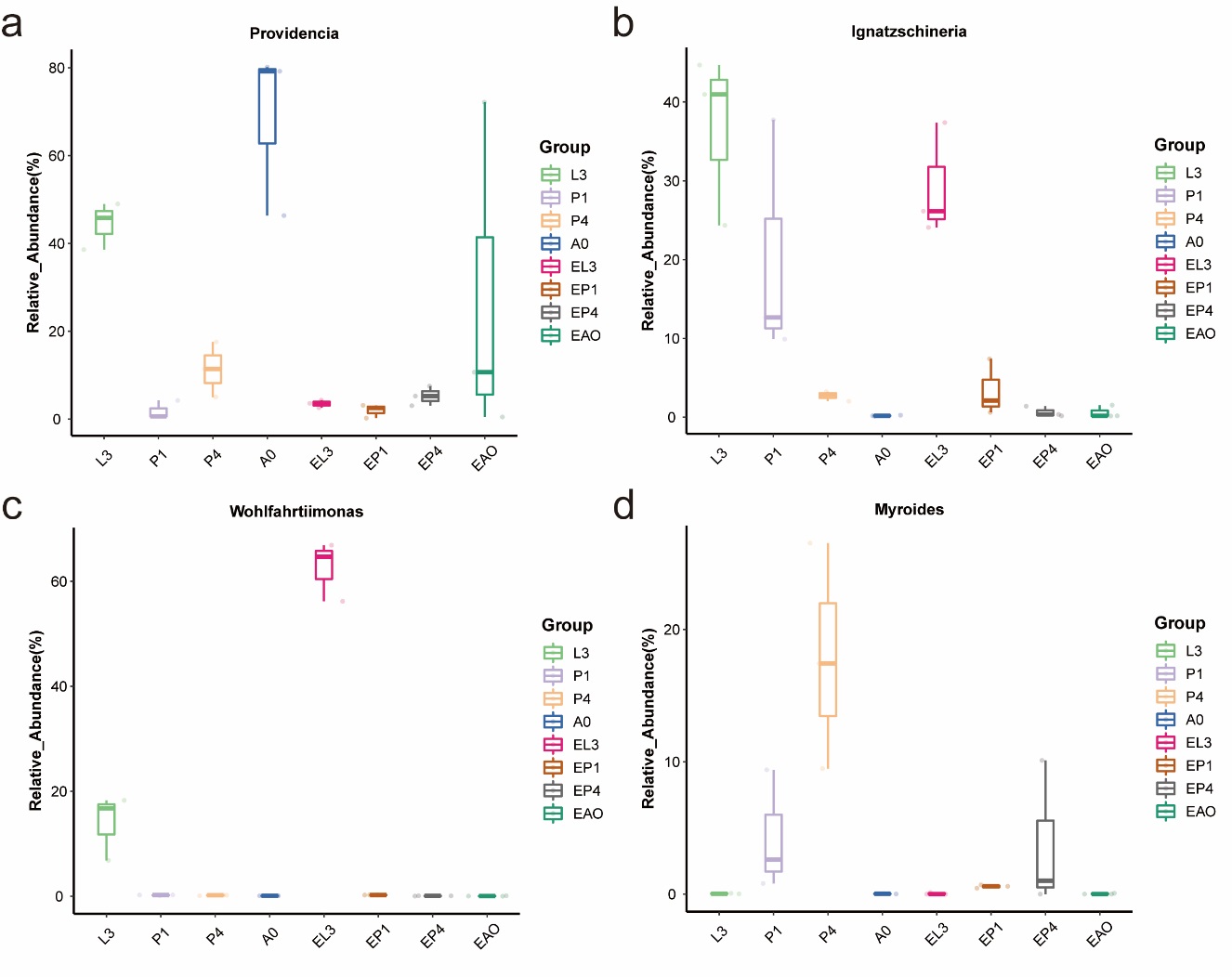


**Fig. S7** The relative abundances of top bacterial flora between heat stress group and the control group analyzed by ANOVA at genus level.
